# fNIRS: An Emergent Method to Document Functional Cortical Activity during Infant Movements

**DOI:** 10.3389/fpsyg.2016.00533

**Published:** 2016-04-20

**Authors:** Ryota Nishiyori

**Affiliations:** Developmental Neuromotor Control Lab, School of Kinesiology, University of MichiganAnn Arbor, MI, USA

**Keywords:** fNIRS, motor development, goal-directed actions, infant reaching, neuroimaging methods

## Abstract

The neural basis underlying the emergence of goal-directed actions in infants has been severely understudied, with minimal empirical evidence for hypotheses proposed. This was largely due to the technological constraints of traditional neuroimaging techniques. Recently, functional near-infrared spectroscopy (fNIRS) technology has emerged as a tool developmental scientists are finding useful to examine cortical activity, particularly in young children and infants due to its greater tolerance to movements than other neuroimaging techniques. fNIRS provides an opportunity to finally begin to examine the neural underpinnings as infants develop goal-directed actions. In this methodological paper, I will outline the utility, challenges, and outcomes of using fNIRS to measure the changes in cortical activity as infants reach for an object. I will describe the advantages and limitations of the technology, the setup I used to study primary motor cortex activity during infant reaching, and example steps in the analyses processes. I will present exemplar data to illustrate the feasibility of this technique to quantify changes in hemodynamic activity as infants move. The viability of this research method opens the door to expanding studies of the development of neural activity related to goal-directed actions in infants. I encourage others to share details of techniques used, as well, including analyticals, to help this neuroimaging technology grow as others, such as EEG and fMRI have.

The depth and range of specific foci in this Research Topic section illustrate that the ontogeny of reaching has been an important area of research in both developmental movement science and psychology. However, the neural basis underlying an infant's production of goal-directed actions has yet to be determined. Scientists in motor development have been yearning for empirical evidence of infant brain activation patterns that support the kinematic and kinetic patterns of functional motor skills. Over the past two decades, functional near-infrared spectroscopy (fNIRS) has emerged as a neuroimaging technique that promises to enable studies of the brain activation patterns in infants. The goal of this paper is to elucidate the utility of fNIRS in the context of goal-directed infant reaching. The first section outlines the knowledge gap in our understanding of neuromotor development and the need to examine brain activation patterns in this field. The following section highlights traditional neuroimaging techniques and how they compare to fNIRS, followed by a brief history and the basic physics of the fNIRS technology. The next section focuses on the processing stream of data that shows the changes in hemodynamic activity of the primary motor cortex as infants reach for an object. Here, the challenges of the processing and analysis data are highlighted. The final section of this paper contains research questions for future studies that will help build broader empirical bases for understanding the central nervous system's (CNS) contributions to the emergence of goal-directed actions.

## The knowledge gap in neuromotor development

How can the direct examination of brain activity during infants' reaching validate or challenge our theories about the emergence of functional motor skills? Theory and data suggest that multiple subsystems contribute to the emergence of first reaches (Thelen et al., 1993; Clearfield and Thelen, 2001). As infants gain sufficient muscle strength, eye-hand visual perception, and self-initiated practice moving their arms, reaching patterns manifest as babies attempt to make hand contact with objects. Further, each of these subsystems has its own developmental trajectory. For example, initially more muscles are activated than “needed,” and infants co-activate muscles to reach for an object (Thelen et al., 1993, 1996). With practice, these movements become smoother and muscle activation patterns become more efficient (Thelen et al., 1993, 1996).

At the CNS level, the theory of neuronal group selection (Edelman, 1987; Sporns and Edelman, 1993) and dynamic neural field theory (Schöner et al., 1997) hypothesize that the brain becomes organized to contribute to the production of successful goal-directed task (Byrge et al., 2014). We do not know, however, how the brain areas associated with goal-directed actions evolve as infants are developing reaching patterns that lead to consistent, sequential, and efficient patterns. The investigation of this unexplored frontier would yield insight onto the ontogeny of brain activation patterns that parallel the development of both the novel skills and improvements in control over these behaviors. Ultimately, such findings are critical to provide foundational understanding and optimize development in those with motor deficits and delays.

Extensive research provides the basis for an adult model of the CNS activity during motor learning and the initiation and control of motor actions. Specific regions, such as the primary motor cortex (M1), prefrontal cortex (pFC), and cerebellum (Crbl) play complimentary and unique roles during different stages of learning (Doyon and Benali, 2005; Halsband and Lange, 2006). The M1 drives neural activations of muscles for voluntary limb movements, the pFC increases activity during the early phases of learning when there are high number of errors, and the Crbl, through feedback processing, adaptively controls the limb movement and trajectory. Adult brains, unlike infants', have years of experience and practice learning to perform new behaviors. Thus, it would be difficult to claim that tasks commonly used in adult brain-imaging studies are truly novel and not simply adaptive. However, we do not know if these same CNS areas play the same roles as infants learn to produce goal-directed actions. We have the technology to verify that infants visually engage with and explore attractive toys prior to reaching (Corbetta et al., 2012), but we do not have evidence of specific brain regions that are activated, or in what sequence they contribute to early and ultimately skilled and adaptive behavior.

## Comparison of techniques

Traditional neuroimaging techniques such as functional magnetic resonance imaging (fMRI) and electroencephalography (EEG) have provided rich information regarding the specific functions and temporal processing of brain regions that underlie motor learning and control. The external validity of these studies can be limited by technical constraints. For example, studies of upper limb motor control in the fMRI scanning environment often involve button presses or reaches with limited degrees of freedom. This limitation is imposed both by the tight space of the scanning environment and the need to reduce noise resulting from head movement. Further, the requirement to lay supine during data acquisition may introduce differential cognitive demands or visuospatial relationships that would not be present in the normative environment. While many adults can cope with environmental and technical constraints the unfamiliarity and noise of the fMRI scanning environment can be unsettling for young children and infants who are required to stay awake and alert during data acquisition. Additionally, infants seldom remain still for extended periods of time and may not have developed the abilities to overcome increased cognitive demands associated with mirrored visual displays or altered visuospatial requirements.

While EEG removes some of the cognitive and visuospatial issues associated with laying supine, degrees of freedom are often still limited to avoid muscular artifacts, ocular artifacts and/or large-scale drifts in the data that result from electromagnetic noise. Strict thresholds for various artifacts result in discarded data during the analyses of infant samples (Stets et al., 2012) leading to the need for high number of trials. Advances in active electrode technology and data analyses have provided some promising results in adult behaviors, such as walking (Gwin et al., 2010) however, these techniques still need improvement (Kline et al., 2015). Set-up times of 1–2 h to prepare the required number of channels place unrealistic expectations on the tolerance of the infant even before any data has been collected. Moreover, the low tolerance to movements across populations in fMRI or EEG limits the type of motor skills that can be investigated. Such technological constraints have held back the field of neuromotor development from making significant progress acquiring the empirical data to confirm hypotheses regarding the neural basis of early motor skill acquisition. Interestingly, however, two studies (Bell and Fox, 1996; Corbetta et al., 2014) have measured EEG coherence, or change in synaptogenesis, and cortical reorganization as infants gained experience with a new motor skill (e.g., crawling or walking). Such studies demonstrate that efforts have been made using EEG to capture developmental changes of the CNS as infants acquire motor skills.

Recently, fNIRS has become a popular tool among developmental scientists to investigate the cortical activation patterns of young children and infants (Vanderwert and Nelson, 2014). fNIRS is a non-invasive neuroimaging technique, which makes it safe to use with infants and can be used repeatedly and for long periods of time. The fNIRS technology and setup allows for larger body movement compared to traditional techniques, making it a particularly effective neuroimaging tool in pediatric research. Furthermore, fNIRS offers improved temporal resolution compared to fMRI and spatial resolution compared to EEG. Moreover, the spatial resolution of fNIRS, although inferior to that of fMRI, affords the ability to localize patterns of activity to specific cortical regions. Such information is critical when investigating the rapidly developing brain of young infants and children.

As a result, the number of researchers using fNIRS to study behaviors and populations that were difficult or nearly impossible with traditional neuroimaging techniques (e.g., fMRI and EEG) have increased substantially (Boas et al., 2014) over the past two decades. Moreover, studies focused on young children and infants have shown the largest increase (Lloyd-Fox et al., 2010; Aslin et al., 2015; Wilcox and Biondi, 2015).

## Emergence of fNIRS

fNIRS was first used as an assessment to monitor the adequate delivery of nutrition and oxygen of the brain in preterm infants receiving intensive care (Brazy et al., 1985). This technique then evolved into clinical studies using single-channel measurements. In 1993, Hoshi and colleagues successfully measured and described neural activity in different areas of the cortex by using five single-channel measurement points (Hoshi and Tamura, 1993). From then, the fNIRS technology developed rapidly and successfully employed multi-channel systems that have provided three-dimensional images (Ferrari and Quaresima, 2012). Specifically, over the past decade, the technique has flourished into a reliable and effective tool to quantify changes in cortical oxygenation in participants across the lifespan.

### How does fNIRS work and what does it measure?

**Figure 1:**
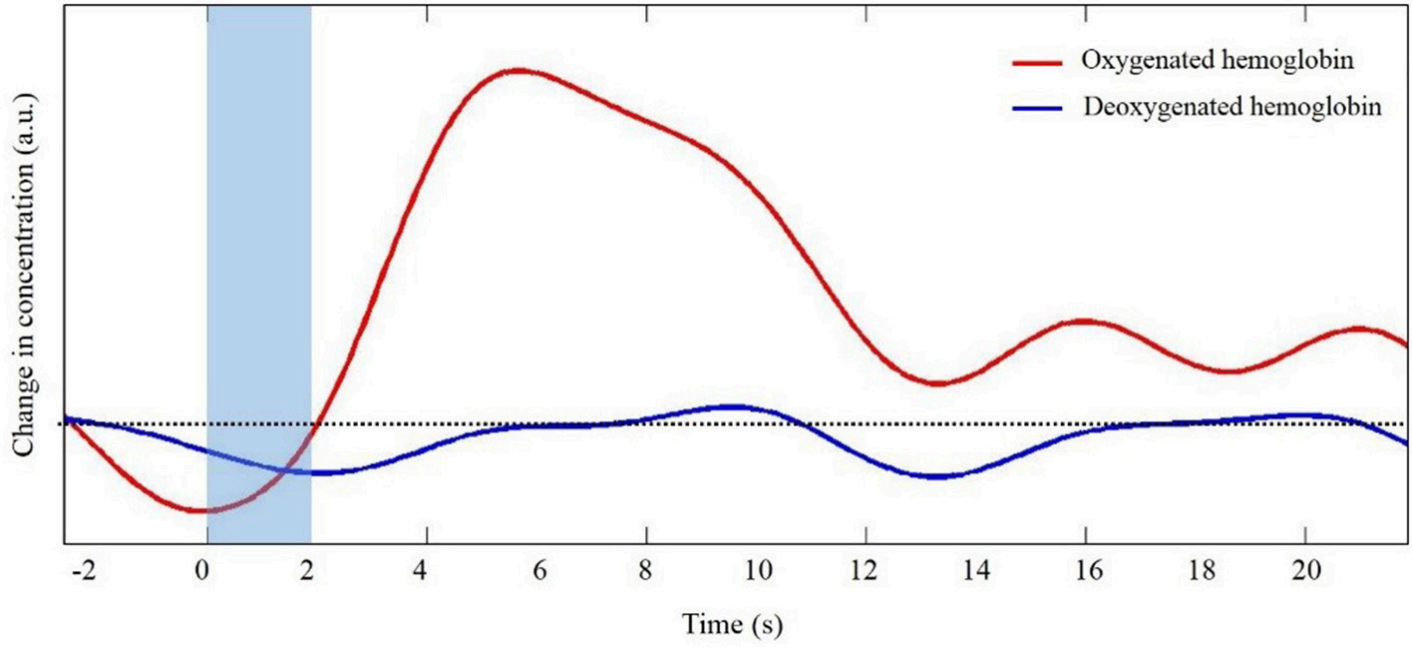
**Representative trace of the canonical hemodynamic response function (HRF)**. Shaded region indicates the time of task. Dotted line indicates zero changes in concentration (i.e., baseline values).

The generation and transmission of electrical activity in neurons is an energy intensive process. When a population of neurons is active, there is an increased metabolic demand. Initially, oxygen supply to the area of neuronal activity lags demand. As oxygen concentration decreases vasoactive agents trigger dilation of local arterioles to increase oxygen rich cerebral blood flow. The influx of oxygen rich blood exceeds oxygen demand such that the local concentration of oxygenated hemoglobin (HbO) increases. At the same time as HbO concentration increases, the local increase in blood flow results in a slight decrease in deoxygenated hemoglobin (HbR). This sequence of events is often portrayed in the form of the canonical hemodynamic response function (HRF, Figure 1). It is this relative difference in HbO and HbR that is quantified to infer changes in neural activity. Unlike fMRI in which concentrations are inferred based upon the different magnetic properties of HbO (diamagnetic) and HbR (paramagnetic), fNIRS takes advantage of differences in light absorption spectra between HbO and HbR.

**Figure 2:**
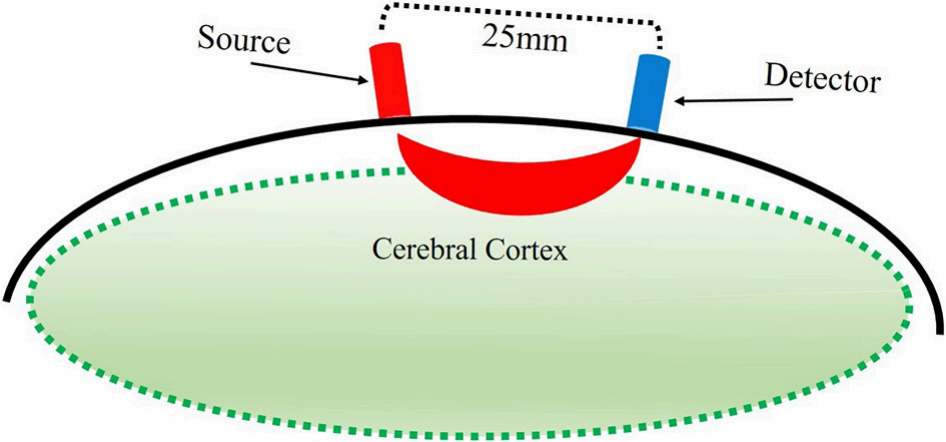
**Illustration of estimated path of near-infrared light between source and detector optodes**.

With fNIRS, near-infrared light is directed via source optodes at the scalp, traveling through the scalp, skull, cerebrospinal fluid, and into the cortical tissue. Light that passes through the cortex is reflected back toward the scalp and is then collected by detector optodes (Figure 2, Villringer and Chance, 1997). Within the near infrared light window (650–1000 nm) of the electromagnetic spectrum, biological tissue is transparent. The light that enters the cortical tissue is predominantly absorbed by hemoglobin. fNIRS utilizes two different wavelengths, each to be sensitive to HbO and HbR. That is, the lower wavelength (650–700 nm), is predominantly absorbed by HbR, while the higher wavelength (800–850 nm) is predominantly absorbed by HbO. The use of two wavelengths allows the calculation of changes in total hemoglobin (HbT). Thus, fNIRS uses the changes in concentration of HbO and HbR as markers of blood flow in the brain to determine function of the area under investigation.

### Overview and setup of fNIRS

The near-infrared light is delivered via fiber optic cables that terminate into a specialized headgear. The optodes can be embedded into the headgear before it is placed on the participant's head, allowing for a much quicker and smoother process to precisely position the cap. This becomes particularly useful when working with infants because repositioning the headgear multiple times can increase the chances of the infant becoming fussy. Once the headgear is in position the fiber optic cables are often bundled or tied into a position that does not interfere with or touch the participant. It is worth mentioning that movement of the fiber optic cables does not introduce artifacts or drifts in the data, which often troubles EEG/ERP studies with young children and infants. Thus, participants can move their head without the introduction of artifacts in the data. This element is particularly useful when measuring infant brain responses, as participants at this age rarely stay still. Energetic and sudden head movements can cause the optodes to move and lose contact with the scalp, leading to artifacts in the data.

The fNIRS technology and setup have limitations as well. The amount and quality of near-infrared light that passes through into the cortex can be affected by large amounts of hair or dark-colored hair that come between the optodes and the scalp. This often leads researchers to devise tightly-fitting caps to ensure the tip of the optodes are as close as possible to the scalp. Alternatively, the hair can be combed away to provide a clear path for the light to pass through the scalp. This issue is a smaller concern with infants as they have not fully developed high volumes of hair.

The use of light and the setup in fNIRS makes it a well-suited tool to investigate the brain activation patterns of infants. fNIRS tolerates perhaps the largest degrees of movement across any neuroimaging technique which invites developmental movement researchers to examine the underlying neural bases of emerging goal-directed actions. To date, however, most studies that use fNIRS with infants and young children investigate visual object processing (Wilcox et al., 2012, 2014) and auditory processing (Gervain et al., 2008; Nakano et al., 2009). There are a few studies that used fNIRS to investigate the motor system as infants observed others performing an action (Lloyd-Fox et al., 2013; Southgate et al., 2014). These studies demonstrate the presence of some form of ability to understand the actions of others. However, these studies do not examine emergent brain activity associated with the inherent control of their own actions requiring online evaluation of sensory feedback and updating of motor plans. Thus, we do not fully understand how the underlying brain activation patterns emerge as infants acquire new functional motor skills. In the next section, the study I will introduce builds on the rich behavioral findings about the ontogeny of reaching and is grounded in strong theoretical framework.

## fNIRS in the context of goal-directed reaching

### Lab setup

**Figure 3:**
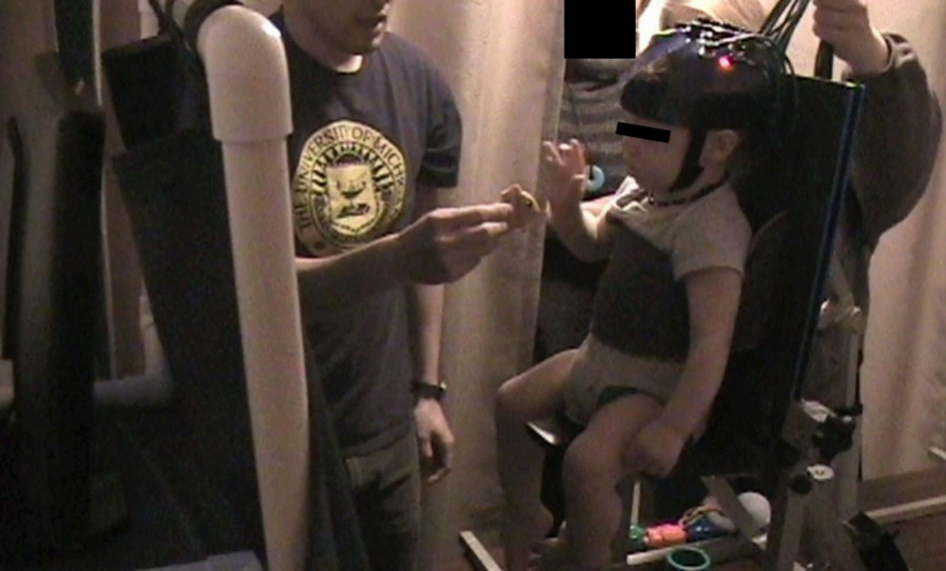
**Picture of lab setup**. Curtain in front of monitor is closed during presentation of toy and reopens after infant reaches for toy during rest phase.

This section presents methodology and unpublished data from a study in the lab, where we measured changes in M1 activity as infants reached for a toy (Nishiyori et al., in press). Briefly, infants were secured in a traditional testing seat used to study infant reaching, with a soft chest wrap to provide security and reduce trunk movement. The seat was on a table so the infant was near the researchers' eye-level (Figure 3). We positioned the headgear, with the optodes already embedded, so that the center of our probe array was directly over the center of the infant's head (Cz, International 10–20 system). The cables extended upward over the infant's head and were held by a research assistant. We positioned a monitor in front of infants who watched calming videos before and after each test trial for no less than 20 s, which allowed us to collect rest-phase values. The experimenter introduced toys within arm reach at midline, and helped keep the infant calm in between test trials.

### Probe array

**Figure 4:**
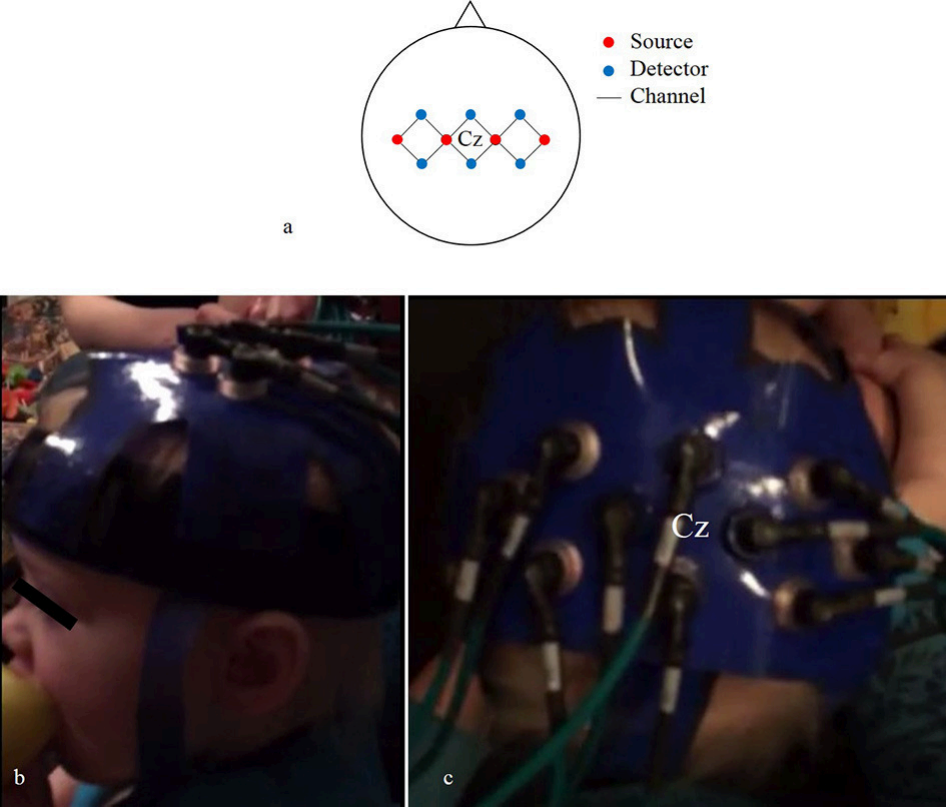
**Probe array (A) of channels (black lines) that connect sources (red circles) and detectors (blue circles)**. Lateral **(B)** and superior **(C)** views of the headgear on an infant's head.

We used four source and six detector optodes, ~25 mm apart, creating 12 channels that covered the bilateral motor cortex (Figure 4A). Each optode terminated into a grommet, a plastic button-like piece that was secured into our headgear (Figures 4B,C). We created our array this way so we could detect hemispheric differences in activity in addition to any bilateral activity. The current adult-based model suggests that contralateral M1 activity drives unilateral limb movements, while bilateral M1 activity drives bilateral limb movements (Nishiyori et al., 2016). Our main question focused on the developmental changes of M1 activity as infants developed functional motor skills. Thus, we wanted to be able to detect patterns of change within the M1 between distinct levels of skill. After several pilot sessions, we decided that 10 optodes (four sources and six detectors) provided the best spatial resolution while bearing the least weight on the infant's head.

### Pre-processing

The data pre-processing stream begins by removing physiological noise (e.g., heart pulsations), slow drifts, and motion artifacts from the optical signal. Low-pass and high-pass filtering are common methods to remove both the physiological noise and slow drifts, respectively. Motion artifacts, on the other hand, are sudden and intense changes in signal and can be removed or corrected using different algorithms. Additionally, we used a digital video recorder to identify different types of behavior offline and during the pre-processing stream.

**Figure 5:**
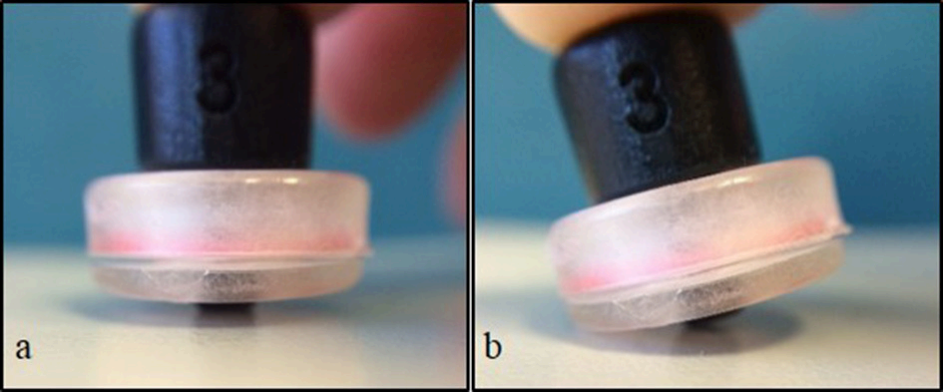
**Simulation of optode position and contact with surface**. Full contact of optode perpendicular to surface **(A)**, and tilt or “jittered” optode caused by movement **(B)**.

We synchronized our video data with our fNIRS data. This enabled us to identify the time of onset of each reach and to determine the amount of movement of uninvolved body segments (e.g., head and legs). There were two types of movements that we were concerned about. The first type was arm and leg movements during the rest phase that could affect our measures. We could then identify and eliminate segments in our fNIRS data that could be affected by these type of movements. The second type are head movements that directly jitter or affect the contact between the tip of the optode and the scalp (Figure 5). When an optode moves and the path of the light is interrupted or redirected momentarily, spikes in the data can be observed. Following a spike, the system may take a few seconds to stabilize. Spikes have high frequencies which are unlike that of biological signals such as hemodynamic responses, and can be easily identified and eliminated in the time series. We were particularly cautious regarding head movements, especially when it was within 2–3 s from the time of onset of reach. The large spikes caused by head movements could influence the amount and intensity of the NIRS measurements. After the noises are removed, the optical signal is converted into concentrations of HbO and HbR using the modified Beer-Lambert Law (Cope et al., 1988).

**Figure 6:**
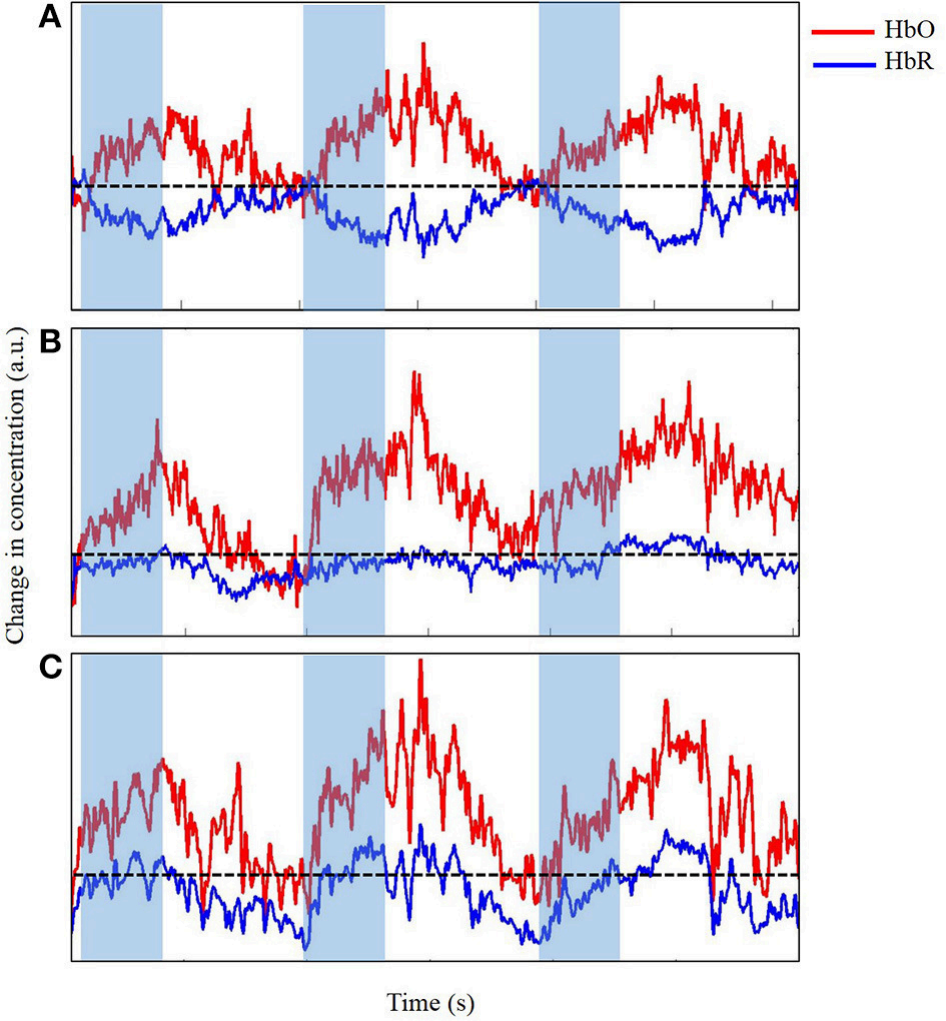
**Time series of change in concentration of Hbo and HbR, unfiltered (A), acceptable (B), and unacceptable (C) data in arbitrary units (a.u.)**. Shaded region indicates time during reach. Dotted line indicates zero changes in concentration.

**Figure 7:**
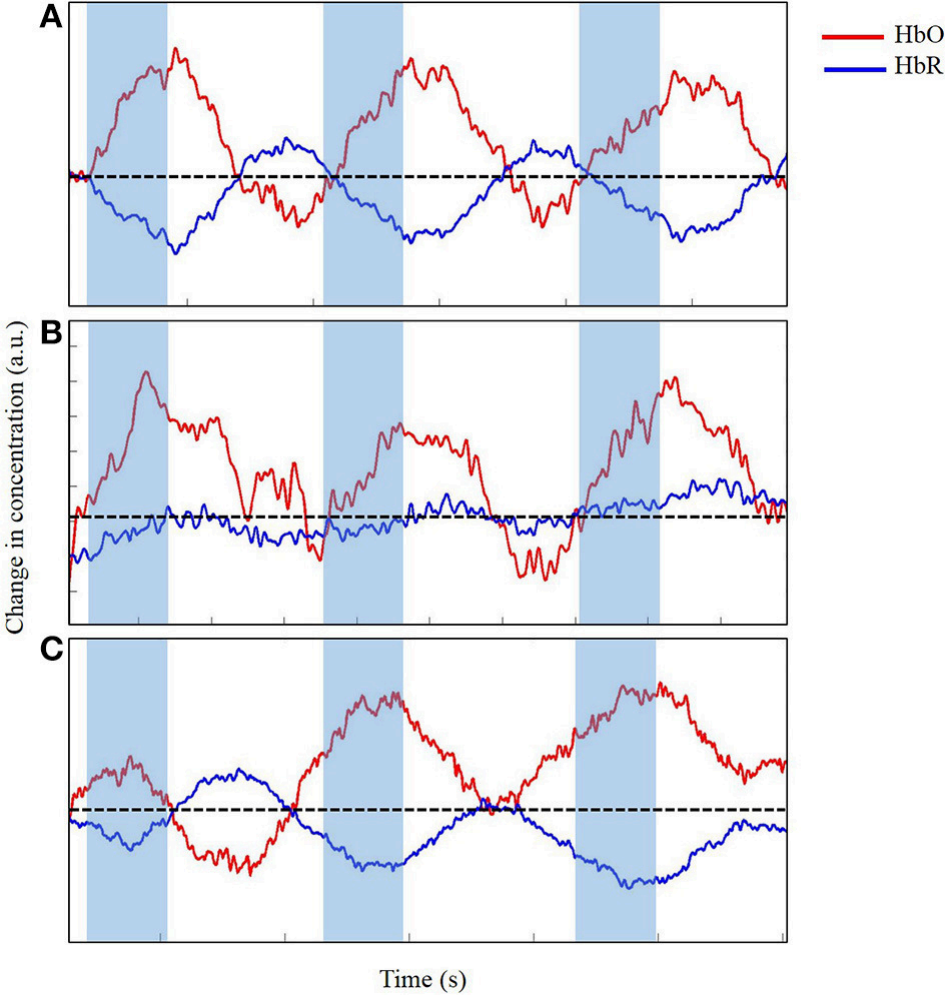
**Time series of change in concentration of HbO and HbR, after wavelet-filtering, optimal (A), acceptable (B) and unacceptable (C) data in arbitrary units (a.u.)**. Shaded region indicates time during reach. Dotted line indicates zero changes in concentration.

We visually examined the time series to accurately determine the changes in concentration of both HbO and HbR in the M1 as infants reached for a toy while eliminating any data contaminated by significant motion artifacts. Figures 6, 7 display portions of the time series from a single channel extracted from the dataset. Each time series represents changes in concentration of HbO and HbR from a single channel. Figure 6A displays a section of the time series that is very clean and in which the expected increase in HbO is easy to identify and clearly timed with the onset of the reach movement. Figure 6B displays a slightly messy time series for a similar reach. The changes in concentration are timed with the onset of the reach, but during the rest-phase, some similar scale increases can be seen. In addition, the increase in HbO also contains small spikes that are caused by extraneous body segment movement, verified through our synchronized video data. This type and frequency of motion-artifacts were the most commonly observed in our dataset (Nishiyori et al., in press). Finally, Figure 6C displays a time series for another reach clearly observed in the video but for which the data would not be considered for further analyses, because most of the time series is contaminated with artifacts caused by jerky head movements. The goal at this stage in pre-processing the data is to eliminate noise, any spontaneous fluctuations, and brain activity that is not tied to the task. The next step is to clean up the data by using, if necessary, motion-correction algorithms to retain trials that may contain a reasonable amount of motion-related artifacts.

The primary goal of motion-correction is to retain as many trials that would otherwise be rejected when it contains motion artifacts. Several approaches have been proposed to assist the filtering process. For example, Virtanen et al. (2011) used an accelerometer to quantify the magnitude of movements to correct for motion artifacts in the fNIRS data. However, additional equipment on an infant's head is not ideal, especially when they already are wearing a cap. Alternatively, most researchers have relied on the changes in the amplitude of the data that is unique to motion-artifacts. This approach can be applied at the post-processing stage by filtering out the motion artifacts. Brigadoi et al. (2014) compared five different algorithms, freely-available, to real functional fNIRS data to correct for motion artifacts. They concluded that correction for artifacts with any of the algorithms retained more trials than simply rejecting trials that contained motion artifacts. Furthermore, the researchers suggested that among the five algorithms they tested, the wavelet filtering (Molavi and Dumont, 2012) retained the most number of trials, making it the most promising technique to correct for motion artifacts (Brigadoi et al., 2014).

In our study, we applied wavelet filtering to best correct our motion-related artifacts. Figure 7 displays the slight improvements of the time series from Figure 6. The time series displayed in Figure 7A shows minimal improvements from Figure 6A because the time series was already clean with minimal artifacts. Figure 7B displays a modest improvement from the slightly messy time series of Figure 6B. The wavelet-filtering proves to be the most effective and useful in this type of time series. Finally, in Figure 7C, the times series has generously improved from Figure 6C. In this case, the motion-correction algorithm is “over-correcting” noise or artifacts in what may be observed as task-related changes in brain oxygenation, and was not considered for further analyses. Particularly for our study, we wanted to distinguish between desired movements (e.g., reaching for the toy) and undesired movements of the leg, trunk, and/or head. Infants reached for a toy, which at times, made them move their bodies and lower limbs. In addition, infants often moved their heads by looking in different directions, which was most likely related to the artifacts we saw in our fNIRS data. Unrelated to the task, fussy infants would move their heads energetically, which introduced the largest artifacts to the data. Thus, during our study, keeping infants content and engaged by funneling their attention to the videos or our research assistants was a crucial step to minimize the number of movement-related artifacts. When infants became fussy briefly, we ran additional trials once the infants calmed down and were relatively content. Sometimes, when infants were looking around the room too often, we extended the rest-phase to ensure a minimum of 30 s in which the infants moved minimally and were relatively calm. These approaches were the product of several pilot sessions that proved to be the most effective while collecting the necessary measures.

Time series similar to that of Figures 7A,B were considered for further analysis. The time series were then epoched, consisting of 3 s prior to and 10 s post-onset of reach. Epochs were then average for each channel and baseline corrected to the pre-movement period (rest). We then compared the changes in HbO and HbR between the two phases, rest and task, to determine significant task-related activity. The location and number of channels, among the 12, that detected task-related activity determined the area or distribution of motor cortex activity during reaching.

Together, with well-designed and piloted equipment set up and motion-correction algorithms, most of the trials from the sessions can be retained. Such movements, both task-related and extraneous, would not be tolerated in most other neuroimaging techniques, but we are able to demonstrate that the fNIRS data is certainly usable and can generate important findings. There are additional challenges that users must be aware of and, we trust will reduce and be eliminated as the technology and software continue to evolve. In the next section, I will touch on a few of these challenges.

## Challenges

### “Rest-phase”

A unique challenge to neuroimaging studies that investigate neuromotor behaviors in infants relates to the need to compare tasks or conditions in order to identify brain activity specifically associated with the test task. Most neuroimaging studies in other domains with infants utilize a large number of trials to calculate the average hemodynamic response for a specific task. For goal-directed actions, however, it is difficult to obtain a high number of trials because infants often do not tolerate repeating the same movement or goal as they express their boredom by failing to attend to the test task. As a result, researchers must determine the number of trials infants will tolerate while also achieving the necessary power to test for significance in task-related change in brain activity. Similarly, the rest phase that precedes the task must also be carefully controlled in order to have a meaningful (usable) trial.

The goal of the rest phase in neuroimaging studies is to allow brain activity to return to baseline or near-baseline. The values measured during the rest phase are often compared with values during the task phase to detect any significant brain activity above baseline. In this setup, the rest phase is important to control in order to detect the task-related changes in brain activity. When rest-phase values contain artifacts or are higher than the task-phase values due to uncontrollable infant behaviors, the comparisons would not allow detection of significant task-related activity. Specifically in our study (Nishiyori et al., in press), we needed infants to be alert while minimally moving any limbs to reduce M1 activity during the rest phase. Our rest-phases consisted of infants watching videos that would keep them alert and minimally engaged. We chose videos that were calming and did not provoke energetic movements.

An alternative approach is to use a control stimulus or task. In this approach, the goal is similar to the static rest-phase, but the values measured during the control task can be used to provide a contrast in brain activity between the experimental task and the control task. Moreover, the control task must be known or hypothesized, a priori, to elicit less brain activity than what is expected of the target brain region during the experimental task. Most often in studies focused on cognitive development, these control tasks are small deviations from the experimental task to target the unique feature inherent to the study or research question (e.g., biological vs. non-biological movement of objects). The challenge becomes finding the best control task for goal-directed actions. For example, the experimenter could present a toy sufficiently out of reach that the infant would only be able to visually explore and not attempt to reach for it. The data acquired during these trials would examine brain activity associated with the observation of the toy and/or the planning of the reaching movement, which should generate additional brain activity. Future users should carefully consider the design of the control task or the use of a static rest-phase to ensure the maximum retention of trials. Ultimately, the control task needs to serve as a comparison/contrast to delineate brain activity associated with the goal-directed action.

An emerging approach that eliminates the need, analytically, for a rest phase involves the examination of differences between HbO and HbR concentrations. This approach, known as correlation based signal improvement (CBSI), is a tool to improve signal quality and delineate functional neural activation. Cui et al. (2010) have suggested using the negative correlation between HbO and HbR to classify the degree of functional neural activation.

The negative correlation is simplistic in design, does not require baseline correction, and is blind to the experimental design, which could improve the signal quality (Cui et al., 2010). CBSI would be able to detect significant activity without the bias of a rest-phase or a control task and has been demonstrated to be effective in functional data with children (Buss et al., 2014) as a method to classify a robust task-related neural response in the underlying cortical regions. CBSI relies on the basic assumption of the canonical hemodynamic response function, in which there is an increase in HbO concentration coupled with a slight decrease in HbR concentration. The correlation, however, may not be as reliable when HbO and/or HbR concentrations asymptote to or overshoot the baseline (Cui et al., 2010).

### Headgear

As most users of the fNIRS technology would agree, the headgear is one of the most essential and crucial pieces of the technology used to acquire a quality set of data. Select fNIRS systems, such as Hitach's ETG-4000 and earlier models, have headgears with pre-determined configurations with set distances (3 cm for adults, 2–2.7 cm for neonates and infants) between source and detector optodes. Other fNIRS systems, such TechEn's CW6 and earlier models, come with free-hanging bundled fiber optic cables. Thus, users can construct the configuration of the optodes. This configuration can be designed, first, by using freely-available software (e.g., SDgui of the AtlasViewer package, Aasted et al., 2015) to precisely map out the positions of each source and detector and how they are interconnected (see Aasted et al., 2015). This enables the user to configure the array into specific shapes with selected distances between sources and detectors depending on the region of interest (ROI) and target population (see Wijeakumar et al., 2015). Furthermore, researchers are establishing methods to digitally register the NIRS probes on an infant MRI template (Lloyd-Fox et al., 2014; Aasted et al., 2015; Emberson et al., 2015). Ultimately, this will allow users to simulate their probe array superimposed on the cortical template to determine if probes cover the intended region(s) of the brain. Next, users must re-create the configuration onto the headgear.

The selection of the headgear's material should be guided by what the target population can tolerate. In most adult studies, headgear is often tight or snug to ensure the tip of the optodes are as close as possible to the scalp. Although this would maximize the likelihood of acquiring data with the fewest motion artifacts, for young children and infants, however, this is often not well tolerated. Thus, users need to choose materials that are infant-friendly but firm enough to hold the optodes in their respective positions. Additionally, the headgear should be easy to fit onto an infant's head to quickly and accurately position it over the ROI, but then be adjustable to assure a snug fit without slippage of optodes away from the intended position.

We found that more traditional fabric or spandex-type caps were too stressful for infants to have put on them and remain on. We also found that using a traditional cap, like a beanie, often left the top, near the vertex, with excess space or creases, which were not desirable for our ROI in the previously mentioned study. The cap, however, may be feasible for measuring other areas such as the frontal and temporal regions. Thus, we used a thin layer of Dycem, a non-slip rubber-like material often used in physical therapy sessions to enhance grip. This material can conform to the different shapes of heads, easy to cut, and rigid enough to hold the grommets. We constructed a two-piece headgear made of Dycem that consisted of a headband and a panel embedded with grommet-pieces for the optodes. The headband had Velcro on the outside and the panel had legs with Velcro pieces on the inside. This allowed us to secure the position of the panel by latching the legs onto the headband. Our headgear proved to be effective, primarily because we only measured motor cortex activity. Studies that investigate multiple areas, especially if the areas are not next to each other, will require several pieces that are connected together.

The goal of the headgear is to secure the optodes in the desired position on the head and to keep the near-infrared light directed at the scalp. Ultimately, the security of optodes will determine how much motion can be tolerated before artifacts are introduced. As a result, users should invest a substantial amount of time designing, constructing, and piloting the headgear.

The two challenges I have outlined here are those that our group has particularly grappled with at the beginning of our study, but also are critical pieces to any new study using the fNIRS technology. There is a community of researchers working together to share the progress in using and processing data, and novel methods. This information is collectively shared at annual workshops and biennial conferences (The Society for Functional Near-Infrared Spectroscopy., 2015). New users can access free resources, such as Homer2 (Huppert et al., 2009) the Matlab based package to preprocess fNIRS data and other tools on the Neuroimaging Informatics Tools and Resources Clearinghouse (NITRC) website as a helpful guide to understand and effectively incorporate fNIRS to answer their research questions.

## Future applications

To the best of our knowledge, we are one of the first groups to explore and examine motor cortex activity in infants as they performed goal-directed actions. The aim was to begin to construct a body of empirical evidence by directly investigating the development of brain activity during functional movements in order to better understand the emergence of and improvement in control of functional motor skills. We began this journey to dig deeper in our understanding of how skills emerge from basic science and theoretical perspective and to provide foundational knowledge that will have clinical applications to optimize development in those with disabilities. To build on this base, we encourage future research to focus on quantifying activity of multiple regions of the brain, sequential activity among regions, longitudinal designs, and assessing the effects of interventions.

### Multiple regions/sequential activity

Goal-directed actions involve volition, planning, and execution, including adapting and correcting, during the movement. For each of these contributions to the behavioral outcome, there are respective brain region(s) involved, each of which develops as the skill emerges over time (Twardosz, 2012; Byrge et al., 2014). Moreover, the amount each region contributes to the action may also fluctuate depending on the level of skill as well as other subsystems that are developing (e.g., executive functions). Future research is needed to determine the changes in neural contributions of different brain regions that underlie goal-directed actions. In addition, the sequence in which each brain region activates leading up to the onset of reach would reveal the unique pattern of activity during early neuromotor control. Such investigations would explore the variability in the way the neural contributions emerge and change across individuals. While the order and contribution of multiple cortical areas in well-practiced skills performed by adults are established, the variability observe during early development, both behaviorally and in the neural data to date, makes a strong case for the theoretical concept, at least in early life, that motor behaviors are softly assembled in response to the demands of the task.

### Elicited vs. voluntary—effects of practice on elicited vs. self-initiated movement

Goal-directed actions are voluntary, but there are many behaviors that can be elicited from an infant. For example, stepping while supported on a treadmill is an elicited behavior that allows researchers to understand behaviors infants can produce without practice (Thelen and Ulrich, 1991). These elicited patterns demonstrate the plasticity and adaptability of the control systems for movement, early in life. Moreover, we know that the CNS in infants undergo significant changes and organization as infants explore and practice ways to control their movements. We do not know, however, which areas or how much each area of the brain changes as the control of movement improves or when or why, from a neural perspective, infants are able to perform elicited patterns such as supported treadmill stepping.

By creating a context in which infants are engaged to practice elicited behaviors, we may help them induce changes at the neural level. These may be distinct from those generated by self-initiated movements. For example, many researchers have constructed unique and clever experiments to address how infants' actions are influenced by their prior experiences. Specifically, Needham and colleagues provided infants ~1 month prior to the onset of reaching, with “sticky mittens” to simulate prehension. The enriched experience showed that infants who gained early experience increased their object engagement and demonstrated more sophisticated object exploration strategies compared to infants with no experience.

Until recently, it was thought that the adhesiveness of the sticky mittens simulated successful grasps, and through repeated experience, goal-directed behaviors were formed (Needham et al., 2002). Williams et al. (2015), however, showed that repeated task exposure with active, reaching-specific experience enhanced formation of goal-directed behaviors compared to grasping simulation through sticky mittens. The later study showed that the task-specific exposure and practice improved goal-directed behaviors more than the simulation of successful reach-and-grasp by sticky-mittens. Comparison of brain organization between task-specific and simulated movements would provide insight to the plasticity of our CNS and how the type or specificity of experience can influence the functional behavior. fNIRS would be a useful tool to shed light on the emerging brain activation patterns as a function of the specific types of experiences.

### Longitudinal designs

To date, most studies investigating brain activity with young children and infants are cross-sectional. In order to understand the organization and reorganization of brain activity, and individual differences in development trajectories, longitudinal designs are necessary. Such designs would provide a better understanding of the reciprocal influences between changes in brain organization and behavioral changes and skill acquisition and control. For example, future researchers can investigate changes in brain activity in the months leading up to and/or months following the onset of successful reaches to determine the changes of motor areas as new functional motor skills emerge.

### Role of the cerebellum

Studies using fNIRS have only been able to successfully quantify cerebral cortex activity; subcortical regions are out of range for the near-infrared light to detect changes in activity because light can only travel a few centimeters through the skull and into the brain tissues (Gervain et al., 2011; Quaresima et al., 2012). The cerebellum (Crbl) is a unique brain structure that is not as deep as subcortical structures such as the amygdala or hippocampus, but in adults the shape of the skull and the cerebellum's position relative to surrounding tissues and neck muscles obstruct the near-infrared light from reaching it for precise measurements. In infants, because the skull's shape is still more rounded and tissues surrounding the cerebellum are much thinner, there is strong reason to believe that the fNIRS technology can be positioned correctly to detect and quantify Crbl activity.

The cerebellum is known for its role in adaptive control and online error correction of targeted movements in adults (Buckner, 2013; Koziol et al., 2014). In infants, the contribution of the cerebellum to motor behavior has been explored minimally. Most hypotheses stem from either theoretical frameworks or data regarding structure and neurophysiology of the cerebellum. In adults, fMRI data show that the cerebellum plays a critical role during the early stages of learning a new skill (Doyon et al., 2002; Halsband and Lange, 2006). Skills that have been tested using fMRI technology, however, are generally deviations or modifications of already well-learned and practiced skills (e.g., finger sequence learning or visual-motor adaptation of manipulandum movement). In other words, the cerebellum needs only to correct or adapt an already-learned motor action. In infants, reaching for a toy is a nascent skill. Infants have been working toward achieving this goal through repeated general movements of the arms often in the direction toward a desired toy, but the “skill” is not yet stable nor functional.

The theory of neuronal group selection (TNGS) proposed by Gerald Edelman suggests that the cerebellum receives sensory inputs and enhances/reinforces successful actions (i.e., the outcome, such as the contact with or grasp of an object) initiated by the motor cortex (Sporns and Edelman, 1993). During development, as infants repeat cycles of acting and perceiving the consequences and persistently try to solve the problem of controlling their limbs, cerebellar activity is hypothesized to be high. The increased activity is later reduced as the accuracy in movement (e.g., arms toward object) improves (Sporns and Edelman, 1993). This hypothesis, is derived from neuroembryology and postnatal neural development data and has been supported via models tested with computer simulations (e.g., Darwin III); it has been further supported via neural monitoring during reaching by monkeys (Georgopoulos et al., 1981), but has yet to be tested directly by measuring cortical activity of human infants. With the emergence of fNIRS, we can measure cerebellar activity during motor learning and test the compatibility between traditional adult studies and infant data to begin to construct an evidence based model of the development of neuromotor control.

## Conclusion

In summary, I have outlined the utility of the fNIRS technology in the context of goal-directed actions. The technology has advantages and limitations; however, it possesses great potential to move the field of neuromotor development forward. fNIRS opens the door to the investigation of brain activity as infants perform motor skills in less-constricted and naturalistic environments. This type of investigation enables researchers to understand the real-time brain activity and its changes over time, as infants improve the control of motor skills. As we continue to identify more clever ways to investigate the development of goal-directed actions, we can expand our knowledge of the brain-behavior link and how it evolves by using the fNIRS technology in future studies. Future users can utilize the information provided here to devise and improve designs to investigate the neural underpinnings of goal-directed actions in infants. Over time, new findings will emerge and we can successfully build the body of empirical evidence that delineates the developmental model, and not infer from the adult-based models of neuromotor control and learning.

## Author contributions

The author confirms being the sole contributor of this work and approved it for publication.

### Conflict of interest statement

The author declares that the research was conducted in the absence of any commercial or financial relationships that could be construed as a potential conflict of interest.
